# Methods for Analysing Patient-Level Utility Data: Insights from the Literature Review and Recent NICE Technology Appraisals

**DOI:** 10.1007/s40273-026-01644-x

**Published:** 2026-09-06

**Authors:** Saul Stevens, Maxwell S. Barnish, Alan Lovell, Frank Grimsey Jones, Jemma Perks, G.J. Melendez-Torres, Elham Nikram, Anthony Hatswell, Elsa Marques, Matthew Franklin, Dan Gallacher, Vanessa Buchanan, Dawn Lee

**Affiliations:** 1 Peninsula Technology Assessment Group (PenTAG), University of Exeter Medical School, Exeter; 2 Petauri Evidence Ltd, Bicester; 3 Evidence Synthesis Group, University of Bristol, Bristol; 4 School of Medicine and Population Health, University of Sheffield, Sheffield; 5 Centre for Evidence and Implementation Science, University of Birmingham, Birmingham; 6 Cogentia Ltd, Cambridge

## Abstract

**Objectives:**

This study examines how patient-level utility data are analysed within NICE Technology Appraisals (TAs) and assesses the extent to which current practice aligns with methodological guidance identified in the published literature. Patient-level utility analysis is critical for estimating health-state utility values used in cost-utility modelling; however, no NICE Technical Support Document currently provides guidance on its appropriate implementation.

**Methods:**

Three complementary reviews were undertaken. A landscape review of the published literature identified recommended methods for analysing patient-level utility data. A targeted review was undertaken to supplement the findings of the landscape review. A review of recent NICE TAs assessed how utility data were collected and analysed, including the methods used, and issues raised by External Assessment Groups (EAGs) and NICE Committees.

**Results:**

Twenty studies met inclusion criteria for the literature review, providing recommendations on core analytical methods but limited guidance on addressing complex data features. Targeted literature searches identified 32 studies, and two further seminal papers were added through expert knowledge. They provided recommendations on approaches to handling repeated measures, missing data, model specification, and covariate selection. The TA review included 125 appraisals. Although mixed-effects models were the most common analytical approach, substantial heterogeneity in methods remained.

**Conclusions:**

While some best practices have been adopted in NICE submissions, variation and limited transparency persist in the analysis of patient-level utility data. The three methodological challenges most frequently identified as issues were capturing sub-response treatment benefits, modelling the long-term evolution of utility beyond progression or treatment discontinuation, and capturing acute disease or treatment features. They are also among the areas least well covered by existing methodological guidance. The development of best-practice guidance for patient-level utility analysis should therefore prioritise these three areas to support more consistent and robust utility estimation for economic evaluation.

## 1 Introduction

In health economic modelling, a health utility is a numerical value that quantifies the preference or value that individuals place on a specific health state, typically measured on a scale where 1 indicates perfect health, 0 represents death and values below 0 indicates states considered to be worse than death. These values are crucial to the health economic modelling process as they are used in the calculation of quality adjusted life years (QALYs), which are made up of life years multiplied by utilities.^1^ A lack of accurate utility inputs can have a major impact on the model’s outcome. This is particularly the case for diseases which primarily impact quality rather than length of life. The introduction of NICE’s severity modifier^2^ further increases the importance of robust utility analysis.

Utility data can come from various sources. They may be generated from patient-level data collected in clinical trials or observational studies using preference-based measures of health-related quality of life (HRQoL) (e.g., EuroQol five dimension (EQ-5D), Short form six dimension (SF-6D), Health Utilities Index (HUI)) or derived through mapping analyses of patient-reported outcomes collected in such studies to preference-based HRQoL instruments.

Once collected, patient-level utility data must be analysed to generate the health-state utility values used in cost-utility models. The objective of this analysis is to translate individual-level HRQoL observations into mean utility estimates that appropriately reflect each modelled health state and the transitions between them. Analytical choices such as the statistical methods, the definition of health states and the approach to handling missing or repeated observations directly determine the utility inputs that drive QALY estimates and the incremental cost-effectiveness ratios. These choices are particularly consequential in oncology, where post-progression utility patterns influence model outputs but are often poorly captured in trial data. They are also consequential in chronic and fluctuating conditions, where repeated measures and long-term trajectories must be estimated from limited follow-up datapoints

NICE guidance specifies a preference for adult utilities to be collected from patients using the EQ-5D-3L or EQ-5D-5L with the UK value set applied, meaning that, where possible, trials should collect such data.^2^

Despite the importance of utilities, there is a major gap in current guidance. No NICE Technical Support Document^3^ provides guidance on how to analyse patient level utility data. As such, utility analysis is an area where much variation is seen in manufacturer submissions.

This review aimed to identify existing best-practice recommendations for the analysis of patient-level utility data and to examine the key issues raised in NICE TAs in relation to current analytical practice. By bringing together methodological guidance from the published literature and critique from recent appraisals, this review provides an initial evidence base to inform the development of best-practice guidance and to support more consistent and robust estimation of health-state utility values in health technology assessment.

## 2 Methods

Two reviews were undertaken. First, a review of the literature to identify best practice recommendations for analysing patient-level utility data and handling key methodological issues. Second, a review of previous TAs to assessed how utility analyses are reported, including methods used and critiques from External Assessment Groups (EAGs) and Committees. The synthesis of issues and best-practice recommendations was conducted using a narrative comparison.

### 2.1 Review #1 (literature review)

#### 2.1.1 Search overview

Given the breadth and heterogeneity of terminology surrounding the topic area, we adopted a two-stage, pragmatic search strategy, consisting of a broad landscape search, followed by targeted searches of specific methodologies. The aim of the landscape review was to identify the scope, key themes, and terminology used across the field rather than to achieve exhaustive coverage.

Based on the landscape review, alongside results from the scoping survey and the ongoing TA review, we identified key themes that warranted deeper investigation. The scoping survey engaged 32 stakeholders from industry and academia to assess the completeness of, and rank, an initial draft list of utility-related issues developed by the project team (full details in the supplementary materials). For these areas, we performed a series of focused, targeted searches to capture additional relevant studies that were unlikely to have been retrieved in the initial broad search due to their specificity. By combining an initial landscape review phase with subsequent targeted searches, we aimed to provide both a high-level overview and nuanced insights into key methodological issues.

#### Landscape search

Searches of the literature were performed in MEDLINE, Embase, PubMed Bookshelf, and the International HTA Database (provided by INAHTA). Only records published after 1997 (the date of the introduction of EQ-5D in the UK) were included. Search strategies are reported in the supplementary materials. We planned to search conference abstracts from ISPOR, HESG, JSM and PSI. However, while it was possible to search ISPOR abstracts via Embase, HESG, JSM and PSI abstracts were unavailable for hand-searching (being either not systematically archived and searchable or not made accessible to non-members).

Complementing the literature search, we requested industry and academic stakeholders (via the scoping survey, detail in supplementary material) to provide relevant publications, code and internal working documents. We also performed citation chasing of relevant systematic reviews identified in the search.

The literature review incorporated a review of the websites of NICE (including their Technical Support Documents) and other key cost-effectiveness HTA bodies for any guidance on the use of patient-level utility data.

#### Sifting and data extraction

Articles were deduplicated in EndNote and screened in Rayyan by a lead reviewer, with independent review of a sample at each stage and disagreements resolved by consensus with a third reviewer. Data extraction was conducted using a standardised form, with a sample independently extracted and discrepancies resolved through discussion.

#### 2.1.2 Targeted searches

Targeted and iterative searches were performed using a combination of Google Scholar, Perplexity.AI and Claude.AI. Potentially relevant articles were identified on screen by the information specialist, who then saved citation details and URLs into an Excel file – records were excluded (i.e. ignored) on screen if already identified by the landscape search. Report authors referred to the Excel file when considering relevant literature during the review process. Further process details and example keywords and prompts are provided in supplementary information.

### 2.2 Review #2 (TA review)

#### 2.2.1 Search and selection

A search of the NICE website was performed to retrieve, and download into Excel, all TAs from the last three years (1 Jan 2022 to 1 Jan 2025). It was decided that three years was a suitable cut-off to ensure a representative sample of recent TAs while also keeping the workload manageable. We included Multiple Technology Appraisals (MTA), Single Technology Appraisals (STA) and Highly Specialised Technology (HST) assessments that used a cost-utility approach. We did not include cost comparison or cost minimisation analyses.

#### 2.2.2 Data extraction

Data extraction was conducted using a standardised form. The following data fields were populated: disease area; utility data source; utility data calculation method; covariates used in calculation; timepoints where utility data were collected; health states used in cost effectiveness model; proportion of missing data in utility calculation; issue(s) identified with calculation or implementation of utility data; issue(s) raised in clarification questions; issue(s) raised in EAG key issues; committee discussion on issue(s); sensitivity analysis performed on utility data andutility values used in cost effectiveness model.

## 3 Results

### 3.1 Search and screening process results

The landscape review identified a total of 3,396 database records and 19 records from supplementary searches. Following de-duplication, a total of 2,414 records were reviewed at title and abstract; 156 articles proceeded to full-text review. Of these, 20 were included in our review of the literature, each a unique study. Among the 136 excluded articles, 16 were systematic reviews that we used to check bibliographies, 113 were excluded on study design, 4 on publication type, 2 on outcomes, and 1 on foreign language. A full list of excluded studies is provided in the supplementary material. Targeted literature searches identified 32 studies, and two further seminal papers were added through expert knowledge. These 34 studies were used to supplement the information gathered from the landscape review. The full process is presented in the PRISMA diagram in supplementary material.

Review #2 identified a total of 289 TAs, with 52 TAs excluded for being terminated appraisals, 4 TAs the documents were not publicly available. 51 TAs excluded as they did not contain utility values and 57 TAs excluded as the utility values presented in the TA were not analysed at the individual patient level. Of the TAs that did not analyse individual patient level utilities, 22 used non patient level data analysis methods such as vignette studies, 26 used utility values taken directly from published literature without further analysis and 9 used utility values from previous TAs without further analysis. These methods were out of scope for this review. There were a total of 164 excluded TAs and 125 included TAs.

### 3.2 Landscape review

There were 20 studies included in the landscape review. The studies provided some good guidance on how to conduct the core stages of utility data analysis, but more limited guidance on how to implement adjustments to address specific characteristics of the data that occur in some cases. The most addressed issue was missing data (11 studies). Other issues addressed were short-term data collection, regression analysis, addressing repeated measures, ceiling effects and the inclusion of covariates including mediator variables. A full narrative description of the literature review and the chief topics it covered can be found in the Supplementary materials.

In summary, the published literature provided helpful guidance in the following areas:

- Ten studies addressed handling missing data and the best way to respond to different patterns of missingness. ^4–12^ The studies examining missing data in economic evaluation largely reflect ongoing debates in the analysis of missing data more generally, though with a stronger preference for simpler imputation methods that would otherwise not be preferred in health research. Methodologists considering analysis of health state utility values showed preference for the use of mixed models to account for repeated measures. Little relevant comment on extrapolation was found in included studies.
- Some studies focused on which regression model to apply. With mixed recommendations, depending on the structure of the data. ^13–16^
- Some studies and reports provided general guidance on how to conduct utility analysis, with some discussion of analysing patient utility data.^17, 18^

There were several gaps in the published literature identified through narrative synthesis of the literature. These particularly related to simulation studies comparing the relative performance of different approaches in different contexts. These include: establishing the degree of floor/ceiling effect that warrants a more complicated model structure to account for these; establishing the optimal regression model under different timepoint structures; establishing the impact of different levels and patterns of missingness and optimal methods to adjust for these;, and understanding the trade-off between the role of missing data and data timepoint structure when determining the optimal regression model, as not all approaches can be combined.

Other key research gaps that were identified from the landscape review narrative synthesis included the best approach to specifying models, particularly selecting and defining covariates, and the best approach when the decision problem population is not fully aligned with the trial population.

### 3.3 Targeted searches

#### 3.3.1 Core components of utility analysis

##### 3.3.1.1 Covariates

Several papers described approaches for selecting and structuring covariates in utility analyses. General principles for specifying, justifying, and assessing covariates within statistical models were outlined by Sanghavi et al. (2024).^19^ Two papers examined covariate structures within time-to-death (TTD) frameworks, where utilities are modelled as a function of time prior to death rather than disease progression status Hatswell et al. (2021)^20^ compared alternative approaches to incorporating TTD into utility estimation using simulation studies, noting the need to examine the structure of the utility dataset and to justify the analytical approach. Hatswell et al. (2024)^21^ applied continuous TTD transformations to empirical oncology trial data, reporting that continuous TTD functions can be appropriate when modelling end-of-life utility patterns.

##### 3.3.1.2 Missing data

Several sources addressed methods for managing missing utility data. Gabrio et al. (2022)^22^ described the use of linear mixed models (LMMs) for handling missing-at-random (MAR) utility data, noting that LMMs operate within a standard regression framework, are deterministic, and can be easily reproduced, offering an alternative to multiple imputation for some users. Leurent et al. (2018)^23^ documented the prevalence and patterns of missingness in trial-based cost-effectiveness analyses and summarised the analytical strategies used, highlighting the importance of reporting missingness clearly, reducing missing data where feasible, and conducting sensitivity analyses rather than defaulting to complete-case analysis. A further study by Leurent et al. (2018)^24^ presented methods for exploring missing-not-at-random (MNAR) mechanisms using pattern-mixture and delta-adjustment approaches, noting that routine assessment of sensitivity to departures from MAR is required to evaluate robustness.

Heymans et al (2022)^25^ stated that complete case analysis is valid where data are missing completely at random (MCAR) and where data are MAR complete case analysis may yield valid results, but in most cases imputation should be used. When imputation is used, multiple imputation should be the method as it leads to valid estimates about the uncertainty of the imputed values.

Similarly, Jakobsen et al (2019)^26^ state that complete case analysis is valid when missing proportions are below 5%, if only the dependent variable has missing values and auxiliary variables and if it is relatively certain that data are MCAR. Multiple imputation should be used when missingness is moderate and MAR is plausible. In cases of large proportions of missing data, complete case analysis should be used and clearly state the limitations of the analysis given the level of missingness.

##### 3.3.1.3 Regression modelling

Several papers discussed regression approaches for analysing utility outcomes. Arslan and Benke (2023)^27^ described considerations for modelling bounded and skewed data, including the use of medians for highly skewed distributions and cumulative distribution functions when presenting uncertainty. Wolowacz et al. (2016)^17^ outlined approaches for handling skewness, excess zeros, and bounded distributions in clinical-study utility data, including transforming utilities to a decrement scale and applying methods commonly used for skewed data such as generalised linear models (GLMs), generalised estimating equations (GEEs), and two-part models in the presence of zero-inflation.

#### 3.3.2 Adjustments for common issues

##### 3.3.2.1 Failure to capture short term disease features

Several papers examined how adverse events (AEs) can influence short-term utility values. Ghabri et al. (2024)^29^ provided methodological guidance for incorporating AE disutilities, including accounting for duration, severity, and AE likelihood, and for applying appropriate methods to quantify their effects on HRQoL.

Lu et al. (2023)^30^ reviewed oncology evaluations and reported variation in AE inclusion and valuation, highlighting the need to determine whether AEs affect HRQoL whether their impact is already reflected in existing utilities, and to specify clear methods for identifying, calculating, and assessing uncertainty in AE disutilities. Pesonen and Kankaanpää (2025)^31^ summarised AE handling in models of pharmacological interventions for diabetes and related conditions, noting the need for further methodological guidance on when and how to include AE effects. Wolowacz et al. (2016)^17^ outlined approaches for capturing short-term utility decrements associated with acute events, including proxy-reported assessments, patient recall of recent AEs, or additional data collection outside the main trial. Brazier et al. (2019)^32^ described considerations for selecting AEs for which utilities are estimated, including incidence, severity, duration, and the expected influence of AE-related utility changes on cost-effectiveness results.

##### 3.3.2.2 Generalisability

Wolowacz et al. (2016)^17^ discussed generalisability considerations for trial-based utility data, including population representativeness, measurement timing, and transferability to economic model contexts. The authors noted that explicit modelling of prognostic factors can support adjustment of trial data to reflect real-world populations more closely.

#### 3.3.3 Sensitivity analysis

Wolowacz et al. (2016)^17^ outlined the use of deterministic and probabilistic sensitivity analyses for utility outcomes, noting the relevance of early economic modelling to assess which utilities warrant high-quality data collection.

Brazier et al. (2019)^32^ described methods for exploring uncertainty in utilities, including one-way and multi-way sensitivity analyses and structured examination of alternative utility sources, emphasising the importance of preserving the logical ordering of utilities across scenarios.

Table 1 presents the best practices recommendations from the literature review process.

### 3.4 Review #2 (TA review)

#### 3.4.1 Characteristics of Utility Evidence Submitted to NICE

##### 3.4.1.1 Types of appraisal and disease area

60% of appraisals evaluated cancer treatments, with the remainder representing non-cancer conditions (40%) and 30% relating to rare diseases. A minority of included conditions were classified as fluctuating (30%). These characteristics indicate that most of the utility evidence reviewed by NICE originates from oncology submissions, with implications for the patterns of data collection and analysis observed across TAs.

##### 3.4.1.2 Sources of utility data and data-collection patterns

Clinical trials were the dominant source of utility data (95%), with only 5% of TAs using non-trial sources such as surveys. In 77% of appraisals, utility data were collected through EQ-5D instruments (EQ-5D-5L and EQ-5D-3L), with cancer trials typically collecting utilities at multiple treatment or disease-related timepoints. Detailed results are provided in Table 2 of the supplementary materials.

Collection frequency varied substantially by disease area. Among TAs reporting timepoints, 92% of cancer submissions collected utilities more than five times during the course of the pivotal clinical trial, compared with 44% of non-cancer TAs. Post-progression data were inconsistently captured: 63% of oncology TAs provided only one utility measurement after progression or discontinuation, and only 14% presented extended follow-up (≥1 year) with regular assessments (≥12 weekly assessments). This was raised in a clarification question by the EAGs on ten occasions and raised as a key issue by the EAGs on eight occasions of the 45 TAs where the clinical data reported only one visit post progression or post discontinuation. Detailed results are provided in Table 3 and Table 4 of the supplementary materials.

##### 3.4.1.3 Transparency and reporting quality

As presented in Table 2, reporting of utility values lacked transparency in a substantial proportion of submissions. Utility values were redacted in 68% of TAs, and 60% did not report the value set used to generate EQ-5D utilities. Additionally, 49% of TAs reported how utilities were incorporated in deterministic sensitivity analysis and 46% of TAs reported how utilities were incorporate in probabilistic sensitivity analyses. Where reported, utilities were generally varied using a beta distribution in PSA and by ±20% in one-way sensitivity analysis (OWSA).

#### 3.4.2 Methods Used to Analyse Utility Data

##### 3.4.2.1 Statistical approaches

Mixed-effects repeated-measures models (MMRM) were the most common analytical method, used in 57% of TAs reporting their method, as presented in Table 3. Simpler approaches such as calculating mean utility values were used in 19% of TAs. Other regression methods, including linear mixed model (LMM), generalised estimating equation (GEE), linear regression, generalised linear model (GLM), segmented regression, and ordinary least square (OLS), appeared infrequently, each representing under 7% of submissions. Five TAs did not report the analytical method applied to patient-level utility data.

##### 3.4.2.2 Covariate selection

Progression status was the most frequently included covariate in the base case analysis, it was used in 33% of included TAs. This is presented in Table 4. Other common covariates included: functional status (30% of included TAs), treatment (28% of included TAs), age (16% of included TAs) and baseline utility (25% of included TAs). Two TAs lacked clear reporting of covariates in the NICE committee papers. Functional status was defined as either a clinical or patient self-reported assessment of their medical condition, for example New York Health Association (NYHA) class or glomerular filtration rate (GFR) level.

##### 3.4.2.3 Health-state construction

In oncology TAs, utilities were most commonly structured around progression status (68%), with some submissions also using time-to-death categories, recurrence or event free survival status, treatment status or response status. Non-cancer TAs predominantly defined health states using functional status-defined disease stages (67%), with smaller proportions using response categories or treatment status. These patterns reflect differences in the underlying clinical evidence and disease trajectories across therapeutic areas.

#### 3.4.3 Issues Identified and Implications for Decision Making

##### 3.4.3.1 Frequency and distribution of issues

Across the 125 included TAs, 204 issues relating to utility evidence were identified. 94% of included TAs contained at least one issue. Of these, 157 issues were raised in clarification questions and 119 were identified as key issues in EAG reports. This pattern highlights that challenges with utility evidence are widespread across NICE submissions.

##### 3.4.3.2 Types of issues raised

Table 5 presents the most common issues identified as part of the TA review. The issues that were identified most often are defined below:

“Failing to capture the long-term worsening of disease” occurs when utility data collection ends at progression or treatment discontinuation, so the gradual utility decline experienced by patients in later disease stages is not reflected in the modelled utility values.

“failing to capture acute disease or treatment features” occurs when the timing or frequency of utility assessments misses short-lived but clinically important events, so their impact on utility is absent from the utility inputs.

“failing to capture sub-response treatment benefits”occurs where utility analysis attributes utility gains only to patients meeting a pre-specified clinical responder threshold, omitting the meaningful symptom or functional improvements experienced by patients whose response falls below that threshold.

##### 3.4.3.3 Committee and EAG alignment

Table 5 presents the alignment of the committee to the company or the EAG on the top six issues identified from the TA review. Where committees commented on an issue as part of the TA, committees frequently preferred the EAGs view on the issue compared to the company perspective on the issue. When failing to capture sub-response treatment benefits, there was no instance where the committee aligned with the company on the issue, in 67% of cases they aligned with the EAG, and in 19% of cases they considered the merits of the EAG and company perspectives for decision making. Notably the committee did not comment on 79% of cases of issues related to timepoints not capturing key acute disease or treatment features but did comment on 86% of cases related to failing to capture sub-response treatment benefits.

**Figure 1:**
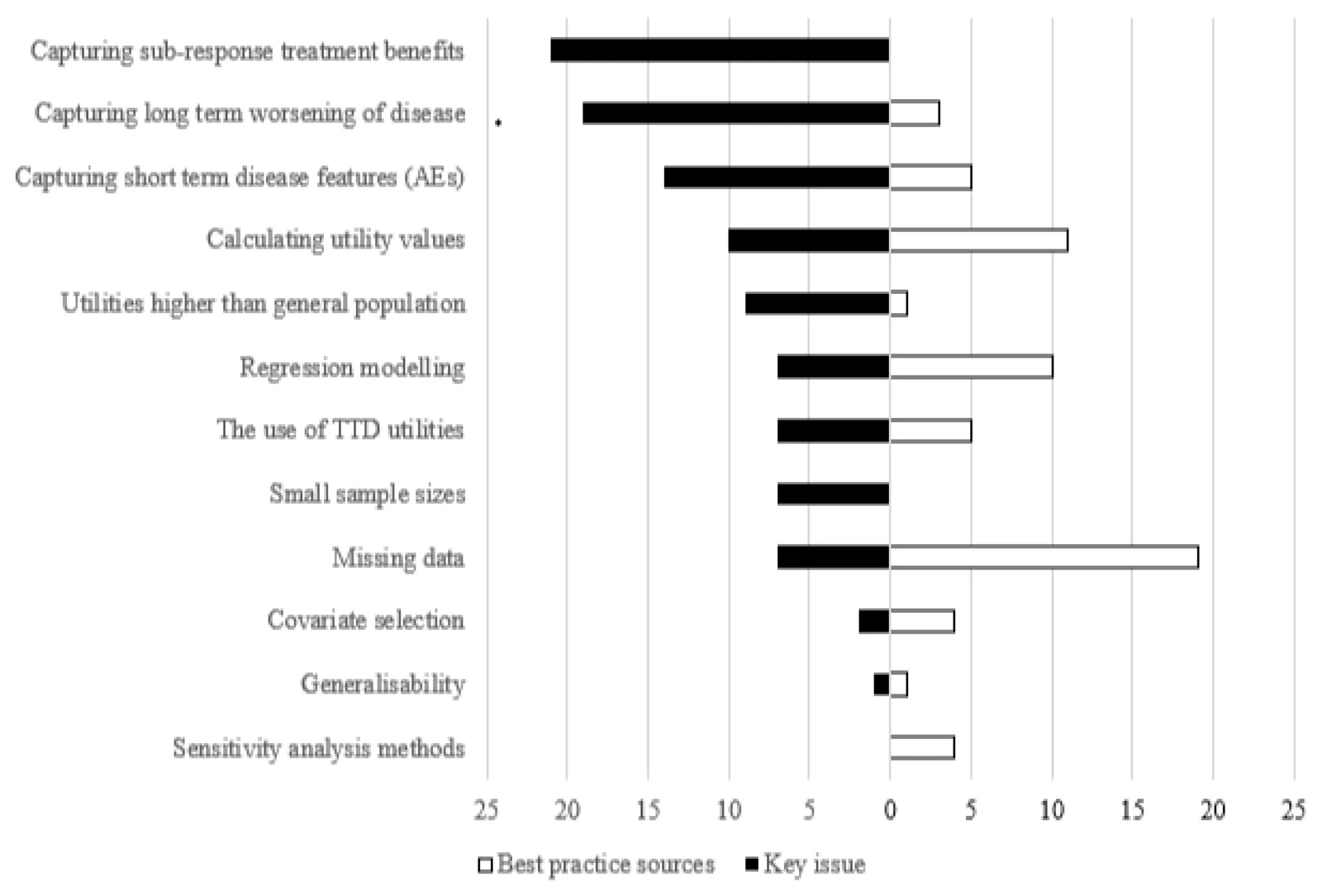
Alignment between issues identified from TAs and recommendations on best practice from literature Abbreviations: AE, adverse event; TA, technology appraisal; TTD, time to death

Figure 1 maps the methodological issues most frequently raised by EAGs in the TA review against the corresponding volume of best-practice guidance identified for each issue through the landscape and targeted literature reviews. The black bars represent the number of times the issue was identified as a key issue by the EAG in the TA review, the white bars represent the number of times guidance on tackling the issue was available in the best practice literature identified in the literature review. The x axis is the frequency, and the y axis are the issues identified from the TA review. The figure is intended to illustrate the degree of alignment between issues encountered in practice and the availability of methodological guidance to address them.

The most prominent issue identified across appraisals relates to capturing the long-term evolution of disease, particularly in oncology. A significant number of oncology TAs (63%) used HRQoL data collection that ceased at treatment discontinuation or disease progression.

## 4 Discussion

This study synthesised published methodological guidance and recent NICE TAs to examine how patient-level utility data are analysed in practice and how this aligns with recommendations from the literature. Overall, converging themes emerged across both evidence streams, highlighting areas of good practice as well as persistent methodological gaps.

It is noteworthy that the three methodological challenges that were most cited by EAGs as key issues (capturing sub-response treatment benefits, long-term worsening of disease, and adverse events) are all areas poorly covered in the methodological literature. First, the limited volume of methodological guidance on appropriate methods to capture the long-term evolution of disease suggests that this challenge remains largely unresolved, despite its repeated impact on decision uncertainty in health technology assessment. Second, not addressing sub-response treatment benefits can lead to systematic underestimation of treatment benefits, particularly for interventions with gradual or heterogeneous effects. The absence of methodological guidance in the literature on how to characterise or quantify sub-response utility gains suggests that this issue has received limited formal attention. Finally, appropriate methods for handling adverse events in utility data analysis are more consistently addressed in the literature, with several sources proposing methods to account for short-term disutility. This may indicate greater methodological maturity in this area, although practical implementation challenges remain. However, absence of clear recommendations on approaches such as borrowing strength from external data or prioritising alternative data sources, presents a methodological gap.

Where well-developed guidance exists, methodological challenges may be more readily anticipated and addressed within submissions. However, as was seen in the TA review, only 14% of TAs reported the proportion of missing data within the NICE committee papers, showing how even where well developed guidance exists, reporting of how or if it is implemented is limited.

## 5 Strengths and Limitations

A key strength of this study is the combined use of evidence from NICE TAs and the published methodological literature, allowing appraisal experience to be examined alongside existing best-practice recommendations. This dual perspective provides insight not only into the frequency of utility-related issues in HTA, but also into the extent to which these issues are supported by available methodological guidance. The structured synthesis of EAG-identified issues with the literature helps to highlight areas of methodological maturity as well as gaps where further research is warranted.

Limitations include that the search approach was necessarily pragmatic, given the topic area’s breadth and heterogeneity of terminology. As a result, it is possible that some relevant publications were not captured, particularly those outside conventional journals. In addition, the review of TAs was restricted to those published within a three-year timeframe.

## 6 Further research

The findings of this review highlight several priority areas where further methodological research is needed to better align health-state utility measurement with the requirements of health technology assessment. There is a need for research that addresses recurrent appraisal concerns which are not currently supported by well-established guidance.

One gap in the published research are simulation studies comparing the relative performance of different approaches in different contexts. Examples of where these studies could provide support are in establishing the optimal regression model under different timepoint structures andproviding a guide on the level of floor/ceiling effects that warrant alternative model specifications.

Further work is required to develop robust approaches for capturing short term disease features, capturing and modelling long term HRQoL and adjusting for sub-response treatment benefits.

## 7 Conclusion

Clearer guidance and further methodological research are needed to support consistent utility estimation. The authors recommend that priority be given to three areas where guidance is currently absent or underdeveloped and where the resulting analytical choices repeatedly drive decision uncertainty:

(i) capturing sub-response treatment benefits
(ii) modelling the long-term evolution of HRQoL beyond progression or treatment discontinuation
(iii) capturing acute disease or treatment features.

These areas are among the least well covered by existing methodological guidance. The authors therefore recommend that the development of best-practice guidance for patient-level utility analysis prioritise these three areas. Addressing them would directly target the issues that most often drive decision uncertainty in NICE appraisals and would meaningfully improve the consistency and robustness of utility evidence in health technology assessment.

## Supplementary Material

Supplementary material

## Figures and Tables

**Table 1:** Best practice recommendations from the literature review

Study	Recommendations
Landscape review	
Anonymous, 2005*^6^	Analyses assuming MAR tend to underestimate HRQoL decline over time where this assumption does not hold.
Bandos et al, 20254	There is no gold standard if outcomes are MNAR. The paper summarises the estimand framework and best practices in stakeholder involvement.
Belger et al, 2016^5^	If the data are MCAR, naive approaches are just as good, but if the data are MAR, MI performs better. All imputation approaches were poor for MNAR.
Blough et al, 20097	Imputation approaches had a substantial impact on the ICER and estimates of uncertainty. The conservative single imputation resulted in less uncertainty. It is recommended to use at least two imputation methods.
Bojke et al, 2017^33^	There is currently no guidance on extrapolating costs and utilities, and little justification for approaches taken. All methods have strengths and limitations. Approaches taken to temporal extrapolation may be too simplistic.
Briggs et al, 2016^34^	As trials are rarely powered to detect differences in HRQoL, a pre-specified regression analysis may be acceptable to improve the precision of the effects and understand their origin.
Faria et al, 2014^8^	Conduct descriptive analysis. Discuss as a team the potential mechanisms for missing data. Mean or MI to impute baseline covariates, MI more complex and may be less efficient but is most widely used. An alternative to MI is likelihood-based models. If the missing data pattern is monotonic IPW methods can be useful.
Griffiths et al, 2017^13^	The mixed regression model explained variation in EQ-5D data according to key clinical outcomes and patient characteristics, providing essential information for long-term predictions of patient HRQoL in the cost-effectiveness model.
Hill et al, 2019^35^	Over half of appraisals applied some form of age adjustment. Committees rarely commented on the limitations of using age-adjusted adult utilities for paediatric populations. While the authors encouraged use of paediatric specific utility measures, where adult measures were to be used, it was recommended that an exchange rate could be agreed for adjustment of utilities.
Hunter et al, 2015^18^	Multiple imputation may overcome the severe limitations of other adjustment approaches. Linear regression with baseline adjustment is a recommended adjustment method.
Kaplan et al, 2005^9^	Imputed measures were significantly correlated with measured utility data but had more constrained variability, showed poor correspondence to ACR20 and ACR50 benchmarks and predicted less than half the variance in actual utility-based outcomes.
Kaproulia et al, 2022^16^	RMME was best in right-bounded and multimodality datasets. The linear mixed model was best in the left-bounded dataset. Across all datasets, OLS, GLM, GEE and tobit performed worst.
Kaproulia et al, 2024^15^	In the event of post-progression drop, this was underestimated by RMME.
Manca et al, 2005^28^	Widely used methods to calculate differential QALY s may provide incorrect results in the presence of baseline mean utility imbalance regardless of whether these differences are formally statistically significant. Multiple regression methods can be usefully applied to generate appropriate estimates of differential mean QALY s and an associated measure of sampling variability, while controlling for differences in baseline mean utility between treatment arms in the trial.
Manca et al, 2005^10^	In one example, the analytical approach did not matter, but in the other results were sensitive to the decision to impute and the approach taken. No recommendations were provided for best practice.
Michalowsky et al, 2020^11^	Item imputation gave a much higher ICER than aggregate imputation.
Pandey et al, 2022^36^	In handling data with heavy ceiling effects, the log-transformed two-part model with OLS was best followed by the log-transformed two-part model with tobit.
Simons et al, 2015^12^	When sample sizes were large and the missing data pattern mainly unit non-response, index or domain imputation produced similar results. If the missing data pattern was mainly item non-response, domain imputation performed better. However, if sample sizes were small, index imputation was preferable
Weston et al, 2013^37^	This is limited by being a conference abstract. It states that the practical considerations of how we should re-analyse trial data for use in an economic model are discussed. However, this detail was only in the full presentation which we cannot access and the abstract does not provide any further detail on the specific recommendations.
Wittenberg et al, 2017^38^	The minimum estimator often performed best when considering the use of joint utility estimators in substance use disorders.
Targeted searches	
Arslan & Benke, 2023^27^	Ceiling and floor effects can be addressed at the experimental design stage by establishing a dose-response curve in a pilot study and adjusting the dose. When there is a significantly skewed distribution, the parametric confidence interval should be replaced by an uncertainty interval derived from the cumulative distribution function. For a highly skewed distribution, the arithmetic mean should be replaced by the median as an estimate of central tendency for the distribution. This article was conducted in the context of economic analysis alongside clinical trials.
Brazier et al, 2019^32^	Decisions should be informed by the relevance of the data and the reliability of the reported analyses. If age-specific health-state utilities are not available, they should be estimated using age-specific population norms. “Condition-free” health state utilities should be estimated from the target population. The extent to which the utility effects of important adverse events are captured by the data used to estimate a model’s non-adverse-event health-state utilities should be assessed. The multiplicative method should be used to represent the utility effects of multiple concurrent clinical events. In the absence of utility data collected during or immediately after an acute (temporary) event, plausible estimates of the QALY loss per event should be used in sensitivity analyses. One-way and multiway sensitivity analyses of health-state utilities should be used. The appropriate order of health-state utilities should be maintained in all sensitivity analyses.
Detry et al, 2016^39^	Mixed models are ideally suited to settings in which the individual trajectory of a particular outcome for a study participant over time is influenced both by factors that can be assumed to be the same for many patients (e.g. the effect of an intervention) and by characteristics that are likely to vary substantially from patient to patient. Using mixed models, reasonably valid estimates of treatment effects can often be obtained even when the missing values are not completely random and additional methods for handling missing data, such as multiple imputation, are generally not required. However, mixed models will have limited validity if their underlying assumptions are not met.
Gabrio et al, *202222*	Linear mixed effects models offer a simple alternative to handle missing data under missing at random without requiring imputation and have not been very well explored in the cost-effectiveness context.
Galofre et al, 2023^40^	This is limited by being a conference abstract. Incorporating time to death in utility analysis may improve utility predictions for advanced oncology indications, particularly when using EORTC-based utilities. The choice of utility analysis method should be carefully assessed since significant heterogeneity exists between indications and data sets.
Ghabri et al, 202429	Improve accounting for adverse events in health state definitions. Provide a transparent framework and documentation of the relationship between adverse events and their likelihood of occurrence, severity, thresholds of severity level, and the health technology. Provide a rationale for identifying adverse events that cannot be captured in the estimates of health state utilities in clinical trials and/or alongside real-world HRQoL studies. Explore novel ways of determining individuals’ preferences related to multiple adverse events and use adequate ways of analysing the impact of adverse events. Report absolute cost effectiveness of adverse events. Characterise structural uncertainty specific to adverse events when reporting the impact of adverse events on health outcomes and cost-effectiveness.
Goldfield et al, 2021^41^	An innovative adaptive Bayesian approach was proposed for pooling patient-level data from several ongoing RCTs that have not been configured as a network of sites and as such the trials share a common active treatment but the control conditions can vary between trials.
Gomer & Yuan, 202342	Multiple imputation via MCMC with a Bayesian linear regression imputation model was the most robust to partial missing-data mechanism violations compared to the other methods considered for a correctly specified regression model with no other assumption violation.
Goncalves et al, 2023^14^	The inconsistent use of methods to account for interdependent observations in the literature indicates that mapping guidelines should include recommendations on how to deal with repeated measurements, and journals should update their guidelines accordingly.
Haliduola et al, 2022^43^	In a missing-not-at-random situation, hybrid imbalanced learning approaches that combine utility-based regression with a synthetic minority oversampling technique for regression in cross-sectional trial settings provides plausible predictions and reduces the bias for realistic missing data scenarios compared to standard approaches such as random forests and multiple imputation.
Hansell et al, 202444	This is limited by being a conference abstract. An assessment of the mechanism and magnitude of missingness should be carried out and reported for all data sets that are used to inform economics models for HTA in the UK.
Harvey et al, 20 1 7^45^	This is limited by being a conference abstract. The role of time-to-death should be considered in future economic evaluations of anti-cancer therapies.
Hatswell et al, 2021^20^	Incorrectly or over-specified utility analysis models can result in substantial bias in the estimation of utilities.
Hatswell et al, 2024^21^	The use of continuous models provides a statistically better fit than time-to-death groupings, without the need for strong assumptions about the health states experienced by patients.
Hatswell et al, 2014^46^	Patient-level utility data should be analysed prior to constructing economic models, as analysis solely by progression status may not capture all predictive factors of patient utility and time-to-death may be important as death approaches.
Leurent et al, 2018^23^	Improvements are required to address missing data in cost-effectiveness analysis conducted alongside randomised trials – focusing on limiting the extent of missing data, choosing an appropriate method for the primary analysis that is valid under contextually plausible assumptions, and conducting sensitivity analyses to departures from the missing-at-random assumption.
Leurent et al, 2018^24^	Cost-effectiveness analysis of randomised trials with missing data should assess the robustness of their findings to possible departures from the missing at random assumption. Multiple imputation provides a flexible and accessible framework to conduct these sensitivity analyses. The results of these analyses should be reported in a transparent way to allow assessment of the plausibility of assumptions made.
Lu et al, 2023^30^	Researchers should attach importance to the impact of adverse events on HRQoL – an application process for adverse event disutilities was presented.
MacNeil Vroomen et al, 20 1 6^47^	Different multiple imputation strategies appeared equivalent with low missingness, but when missingness exceeds 35%, it is recommended to use multiple imputation with predictive mean matching on log-transformed costs.
Morris et al, 2022^48^	No single method for covariate adjustment is always best – the choice will depend on the trial context. Greater use of all three methods was encouraged – direct adjustment, standardisation, and inverse-probability-of-treatment weighting.
Pesonen & Kankaanpaa et al, 2025^31^	Incidence, condition and the measure of utility were all associated with the results being sensitive to adverse drug events. As adverse drug events are an important outcome for the results of economic evaluation, better guidance on their inclusion and exclusion is needed.
Qian et al, 2025^49^	Multiple linear regression is recommended as a simple and universal statistical method for the analysis of patient-reported outcomes in randomised trials.
Sanghavi et al, 2024^19^	Clearly document and justify the analysis and note which aspects were pre-specified. Be transparent about any departures from prespecified methods and provide a rationale. Use prior knowledge, scientific judgment, and mechanism-based models to overcome challenges arising from inevitable correlations of covariates, such as size, age, and organ function. Ensure the base model is stable and consistently estimated. Screening (data-driven) approaches may be satisfactory when prediction is the primary goal. If the model is to be used for identifying covariate relations that may be important in dosing or for other descriptive purposes, a pre-specification approach will generally be the most appropriate. Use sensitivity analyses as necessary to ensure that the covariate model is not unduly affected by parametric assumptions or outliers. Facilitate the proper interpretation of negative findings by providing confidence intervals where possible.
Sanghera & Coast, 20 20^50^	When constructing a model in conditions where health fluctuates, consider whether the condition or treatment causes fluctuations and whether they are predictable; whether the outcomes are measured appropriately; whether the methods of analysis could influence the results; and whether the decision model accounts for fluctuations.
Sanghera et al, 2022^51^	The use of patient reported HRQoL measures in fluctuating conditions should be questioned as participants are unable to provide meaningful responses due to the recall period and timing of assessment.
Spencer & Robinson, 20 07^52^	As utility independence holds in the majority of cases examined, its use is generally supported to allow the derivation of more tractable models.
Van Wijk et al, 2023^53^	Individual-patient data meta-analysis is a powerful method for knowledge integration, leveraging systematic review of databases and literature, detailed data at the individual participant level, and quantitative modelling of pharmacokinetic processes, including capturing heterogeneity of variance between studies.
Versteegh et al, 2022^54^	In oncology settings, time-to-death is a predictor value for HRQoL which allows models that lack HRQoL data to extrapolate overall survival estimates. Compared to an annual age-related disutility, the time-to-death approach generates more incremental QALY s, most notably where the survival period is fairly long.
Witte & Didelez, 2019^55^	While there are considerable differences between covariate selection approaches, none is uniformly best. The optimal choice depends on confounding structure as well as the chosen adjustment method. Prior knowledge of the structure of causal relations underpinning the model helps choose the most appropriate method.
Wolowacz et al, 2016^17^	Recommendations are made for early planning of health-utility data collection within a research and development program; design of health-utility data collection during protocol development for a planned clinical trial; design of prospective and cross-sectional observational studies and alternative study types; and statistical analyses and reporting. Specifically, in the context of statistics and reporting, it was recommended to i) use a data analysis approach that reflects the needs of the economic analysis and is not constrained by the traditional approach for analyzing data for regulatory purposes (e.g. comparison between treatment arms), ii) data are constrained to values <1 and are left-skewed – consider a simple linear transformation (X = 1 – U) and then use familiar methods for right-skewed data, e.g. generalized linear modelling or generalised estimating equations for longitudinal data, with the possibility of a two-part model to handle excess zeros, iii) consider explicit modelling of prognostic factors – this has the potential to increase the generalizability of results by adjusting clinical trial data to the characteristics of populations in routine clinical practice, iv) regarding the transferability of data in multinational studies – there are predictable differences in the way patients map to the index score of the utility instrument; country or region effects can be handled as a covariate in statistical modelling; use the country value set or tariff that is appropriate for the economic model’s audience, v) careful reporting can maximise the value of research for future economic evaluations, vi) covariance can be used to retain the integrity of the logical ordering of health-utility estimates under conditions of uncertainty – alternatively consider specifying a functional form that maintains the logical ordering of health state utilities. This is important for other analysts seeking to apply the results in future models, including appropriate assessment of uncertainty.
Wright et al, 20 09^56^	No gold standard method for measuring temporary health states for cost-utility analyses emerged. Method choice will depend on which of these is considered most important: accurately capturing the unique aspects of the temporary health state, level of respondent burden and cognition, theoretical consistency of elicited preference values with the overall aim of the study, and resources available for study development and data collection.
Expert knowledge	
Heymans 2022^25^	Complete case analysis is valid where data are missing completely at random. Where data are missing at random, in some situations a complete case analysis may yield valid results. However, in most situations, missing data imputation should be used. When imputation is used, it is highly advised to use multiple imputation as this leads to valid estimates including about the uncertainty of the imputed values.
Jakobsen 2017^26^	A flowchart is presented. Analysis of complete case data (without multiple imputation) is valid when i) proportions of missing data are below 5% and it is implausible that certain patient groups specifically are lost to follow-up in one of the compared groups, ii) if only the dependent variable has missing values and auxiliary variables (i.e. variables not included in the regression analysis, but correlated with a variable with missing values and/or related to its missingness), iii) if it is relatively certain that the data are missing completely at random. If large proportions of data are missing, it should be considered to just report the complete case analysis and clearly discuss the interpretative limitations of the trial results. Using multiple imputation in the presence of large proportions of missing data might indicate that the trial results are confirmative, which they are not if missingness is considerable. If missing at random is implausible, the trial results will be at risk of bias due to ‘incomplete outcome data bias’ and no statistical method can with certainty take account of this potential bias. The validity of approaches to handle missing not at random require assumptions that cannot be tested based on observed data. In this situation, best-worst and worst-best case sensitivity analysis may show the full theoretical range of uncertainty and conclusions should be related to this uncertainty. If the outcome variable with missing values is continuous and the analytical model is complicated (i.e. has interaction terms), the use of the maximum likelihood method may be suggested to avoid the problems of model compatibility between the analytical model and the multiple imputation model where the former is more general than the latter. If none of the above reasons for not using multiple imputation apply, then multiple imputation could be used.

* Based on Fairclough et al (2023), but this methods paper has no authors. Abbreviations: ACR, American College of Rheumatology; EORTC, European Organisation for Research and Treatment of Cancer; GEE, generalised estimated equation; GLM, generalised linear model; HRQoL, health related quality of life; HTA, health technology assessment; ICER, incremental cost effectiveness ratio; IPW, inverse probability weighting; OLS, ordinary least squares; MAR, missing at random; MCAR, missing completely at random; MCMC, Markov chain monte carlo; MI, multiple imputation; MNAR, missing not at random; QALYs, quality adjusted life years; RCT, randomised controlled trial; RMME, repeated measures mixed effects

**Table 2:** Characteristics of utility evidence submitted to NICE

Characteristic	Yes n (%)	No n (%)
Cancer appraisal (Y/N)	76 (60%)	49 (40%)
Rare disease (Y/N)	38 (30%)	87 (70%)
Fluctuating condition (Y/N)	36 (29%)	89 (71%)
Trial data source (Y/N)	119 (95%)	6 (5%)
Redacted utilities (Y/N)	84 (67%)	41 (33%)
Value set reported (Y/N)	51 (41%)	74 (59%)
DSA method reported (Y/N)	61 (49%)	64 (51%)
PSA method reported (Y/N)	58 (46%)	67 (54%)

Abbreviations: DSA, deterministic sensitivity analysis; PSA, probabilistic sensitivity analysis

**Table 3:** Analytical methods for utility analysis found in NICE TAs

Method	Frequency
Mixed effects repeated measures*	75 (61%)
Simple mean	29 (24%)
Linear regression	7 (6%)
Generalised estimating equation	6 (5%)
Ordinary least squares	3 (2%)
Tobit model	2 (2%)
Generalised linear model	1 (<1%)
Total reported methods	123

Abbreviations: TA, technology appraisal

* Note: two TAs combined generalised linear model and mixed effects with repeated measures and have been classed in this table as mixed effects repeated measures

**Table 4:** Covariates used in base case analysis of NICE TAs

Covariates	Frequency
Progression status	41 (16%)
Functional status	38 (15%)
Treatment	35 (14%)
Baseline utility	31 (12%)
AE status	19 (7%)
Age (type unspecified)	13 (5%)
Timepoint	11 (4%)
Response status	11 (4%)
Gender	9 (4%)
Time to death	8 (3%)
Recurrence or event free survival	8 (3%)
Age continuous	5 (2%)
On/off treatment	4 (2%)
Interaction: treatment*progression status	5 (2%)
Geographical area	3 (1%)
Age grouped	2 (1%)
Interaction: time*treatment	2 (1%)
Other*	9 (4%)
Total covariates used in base case analysis	254

Abbreviations: AE, adverse event; TA, technical appraisal

* Ethnicity, study, prior treatment, time since diagnosis, treatment*response interaction, functional status*functional status interaction, AE status*treatment interaction and progression*response interaction each had one utilisation.

**Table 5:** Issue frequency and committee alignment from NICE TAs

Issue	Frequency	Raised in CQ by EAG (%)	Raised in KI by EAG (%)	Committee alignment with EAG (%)	Committee alignment with company (%)	Committee neutral (%)	Committee no direct comment (%)
Failing to capture long term worsening of disease	32	25 (78%)	19 (59%)	7 (37%)	3 (16%)	2 (11%)	7 (37%)
Failing to capture sub-response treatment benefits/treatment related utilities	24	19 (79%)	21 (88%)	14 (67%)	0 (0%)	4 (19%)	3 (14%)
Timepoints not capturing key acute disease/treatment features (AEs)	22	17 (77%)	14 (64%)	2 (14%)	0 (0%)	1 (7%)	11 (79%)
Utility higher than gen pop	17	12 (71%)	9 (50%)	4 (44%)	1 (11%)	0 (0%)	4 (44%)
Missing data	16	12 (75%)	5 (31%)	0 (0%)	0 (0%)	2 (40%)	3 (60%)
Mapping and instrument selection	14	12 (86%)	10 (77%)	4 (40%)	2 (20%)	1 (10%)	3 (30%)

Abbreviations: AE, adverse event; CQ, clarification question; EAG, external assessment group; KI, key issue; TA, technology appraisal;

## Key points for decision makers

- Patient-level utility data analysis is a critical driver of cost-effectiveness results in NICE Technology Appraisals, yet no NICE formal guideline currently provides guidance on its implementation.
- The most common methodological challenges were the most frequent sources of decision uncertainty raised by External Assessment Groups and the areas least well supported by existing methodological guidance.
- Development of best-practice guidance for patient-level utility analysis should prioritise the methodological challenges, supported by simulation studies comparing analytical approaches across contexts, to improve the consistency of utility evidence informing health technology assessment decisions.
