# Supplementary material for: Methods for Analysing Patient-Level Utility Data: Insights from the Literature Review and Recent NICE Technology Appraisals
Source: Pharmacoeconomics. Author manuscript; Available in PMC 2026 Nov 4. (PMC7619695; doi:10.1007/s40273-026-01644-x)
Supplement: Supplementary file 1 [file EMS218208-supplement-Supplementary_material.docx]

TA & lit review manuscript supplementary material

## Table of contents

[Search strategies	2](#_Toc217024801)

[Inclusion criteria	6](#_Toc217024802)

[PRISMA for literature review search	7](#_Toc217024803)

[PRISMA for previous TA search	8](#_Toc217024804)

[Scoping Survey	9](#_Toc217024805)

[Landscape review: Sifting and data extraction	9](#_Toc217024806)

[Targeted searches: methods detail	10](#_Toc217024807)

[TA review methods detail	11](#_Toc217024808)

[TA review results tables	11](#_Toc217024809)

[Narrative description of key themes in Review #1 (landscape review)	13](#_Toc217024810)

[HTA bodies searched	16](#_Toc217024811)

[List of studies excluded from the systematic review	17](#_Toc217024812)

[Included TAs	26](#_Toc217024813)

[Excluded TAs	29](#_Toc217024814)

[References	34](#_Toc217024815)

# Search strategies

Database(s): **Ovid MEDLINE(R) ALL** 1946 to April 03, 2025
Search Strategy:

| # | Searches | Results |
| --- | --- | --- |
| 1 | quality-adjusted life years/ | 17651 |
| 2 | *"Quality of Life"/ | 125802 |
| 3 | ((health* or patient* or score* or assessment* or value* or measur* or individual*) adj5 utilit*).ab,ti,kw. | 35193 |
| 4 | ("time trade-off" or "time tradeoff" or "time trade off" or "willingness to pay" or "willingness-to-pay" or "standard gamble").ab,ti,kw. | 12484 |
| 5 | (EuroQOL or "eq 5d*" or "eq5d*" or "eq-5d*" or "short form 6d*" or "Short Form 6-Dimension" or "Short-Form 6-Dimension" or "Short-Form 6 Dimension" or "Short Form 6 Dimension" or "short-form 6d*" or "sf6d*" or "sf 6d*" or "sf-6d*" or "Health utilities index" or "HUI-2*" or "HUI2*" or "HUI-3*" or "HUI3*" or "EORTC-8D" or "AQoL*" or "EORTC-8D*" or "ICECAP*" or "QWB*").ti,kw. | 3822 |
| 6 | or/1-5 | 180354 |
| 7 | Cost-Benefit Analysis/mt [Methods] | 2991 |
| 8 | Cost-Effectiveness Analysis/mt [Methods] | 3 |
| 9 | exp *Methods/ | 74591 |
| 10 | *Research Design/ | 45229 |
| 11 | exp *Data Interpretation, Statistical/ | 14911 |
| 12 | *uncertainty/ | 4921 |
| 13 | ((method* or instrument* or tool* or measure* or statistic*) and (utilit* or (quality adj2 life) or (patient adj report*))).ti. | 14614 |
| 14 | (((long* or extend*) adj ("follow-up" or "follow up")) or "missing data").ti. | 3666 |
| 15 | ((extrapolat* or regression) adj2 (data or utilit*)).ti. | 197 |
| 16 | or/7-15 | 113053 |
| 17 | ("patient-level" or "individual-level" or ((individual* or patient* or trial* or registr*) adj3 (level* or data or utilit* or quali* or qaly))).ab,ti,kw. | 670365 |
| 18 | 6 and 16 and 17 | 1672 |

Database(s): **Embase** 1974 to 2025 April 03
Search Strategy:

| # | Searches | Results |
| --- | --- | --- |
| 1 | quality adjusted life year/ or *"quality of life"/ | 193954 |
| 2 | utility value/ | 1576 |
| 3 | ((health* or patient* or score* or assessment* or value* or measur* or individual*) adj5 utilit*).ab,ti,kw. | 54900 |
| 4 | ("time trade-off" or "time tradeoff" or "time trade off" or "willingness to pay" or "willingness-to-pay" or "standard gamble").ab,ti,kw. | 18248 |
| 5 | (EuroQOL or "eq 5d*" or "eq5d*" or "eq-5d*" or "short form 6d*" or "Short Form 6-Dimension" or "Short-Form 6-Dimension" or "Short-Form 6 Dimension" or "Short Form 6 Dimension" or "short-form 6d*" or "sf6d*" or "sf 6d*" or "sf-6d*" or "Health utilities index" or "HUI-2*" or "HUI2*" or "HUI-3*" or "HUI3*" or "EORTC-8D" or "AQoL*" or "EORTC-8D*" or "ICECAP*" or "QWB*").ti,kw. | 5397 |
| 6 | or/1-5 | 249111 |
| 7 | *methodology/ | 25740 |
| 8 | *statistical analysis/ | 21489 |
| 9 | "patient coding"/ | 34966 |
| 10 | *uncertainty/ | 9906 |
| 11 | ((method* or instrument* or tool* or measure* or statistic*) and (utilit* or (quality adj2 life) or (patient adj report*))).ti. | 20584 |
| 12 | (((long* or extend*) adj ("follow-up" or "follow up")) or "missing data").ti. | 5019 |
| 13 | ((extrapolat* or regression) adj2 (data or utilit*)).ti. | 231 |
| 14 | or/7-13 | 116717 |
| 15 | ("patient-level" or "individual-level" or ((individual* or patient* or trial* or registr*) adj3 (level* or data or utilit* or quali* or qaly))).ab,ti,kw. | 1077019 |
| 16 | 6 and 14 and 15 | 2494 |
| 17 | limit 16 to yr="1997 -Current" | 2414 |
| 18 | conference abstract.pt. | 5435986 |
| 19 | 17 not 18 | 1530 |
| 20 | ispor.nc. | 59448 |
| 21 | 17 and 20 | 172 |
| 22 | 19 or 21 | 1702 |

**PubMed Bookshelf**

***Restricted to “books”***

((EuroQOL[Title] OR "eq 5d*"[Title] OR "eq5d*"[Title] OR "eq-5d*"[Title] OR "short form 6d*"[Title] OR "Short Form 6-Dimension"[Title] OR "Short-Form 6-Dimension"[Title] OR "Short-Form 6 Dimension"[Title] OR "Short Form 6 Dimension"[Title] OR "short-form 6d*"[Title] OR "sf6d*"[Title] OR "sf 6d*"[Title] OR "sf-6d*"[Title] OR "Health utilities index"[Title] OR "HUI-2*"[Title] OR "HUI2*"[Title] OR "HUI-3*"[Title] OR "HUI3*"[Title] OR "EORTC-8D"[Title] OR "AQoL*"[Title] OR "EORTC-8D*"[Title] OR "ICECAP*"[Title] OR "QWB*"[Title] OR "time trade-off"[Title] OR "time tradeoff"[Title] OR "time trade off"[Title] OR "willingness to pay"[Title] OR "willingness-to-pay"[Title] OR "standard gamble"[Title] OR "health utility"[Title] OR "patient utility"[Title] OR "health utilities"[Title] OR "patient utilities"[Title]))

**INAHTA**

***Search 1***

(quality-adjusted life years or quality of life)[mh] AND (Methods or Research Design or Data Interpretation, Statistical or uncertainty)[mh] AND (("patient-level" or "individual-level" or ((individual* or patient* or trial* or registr*) and (level* or data or utilit* or quali* or qaly))))

**Search 2**

(("patient-level" or "individual-level" or ((individual* or patient* or trial* or registr*) and (level* or data or utilit* or quali* or qaly)))) AND (((health* or patient* or score* or assessment* or value* or measur* or individual*) and utilit*) OR ("time trade-off" or "time tradeoff" or "time trade off" or "willingness to pay" or "willingness-to-pay" or "standard gamble") OR (EuroQOL or "eq 5d*" or "eq5d*" or "eq-5d*" or "short form 6d*" or "Short Form 6-Dimension" or "Short-Form 6-Dimension" or "Short-Form 6 Dimension" or "Short Form 6 Dimension" or "short-form 6d*" or "sf6d*" or "sf 6d*" or "sf-6d*" or "Health utilities index" or "HUI-2*" or "HUI2*" or "HUI-3*" or "HUI3*" or "EORTC-8D" or "AQoL*" or "EORTC-8D*" or "ICECAP*" or "QWB*")) AND (Methods or Research Design or Data Interpretation, Statistical or uncertainty)[mh]

**Search 3**

(("patient-level" or "individual-level" or ((individual* or patient* or trial* or registr*) and (level* or data or utilit* or quali* or qaly)))) ) AND (((((health* or patient* or score* or assessment* or value* or measur* or individual*) and utilit*) OR ("time trade-off" or "time tradeoff" or "time trade off" or "willingness to pay" or "willingness-to-pay" or "standard gamble") OR (EuroQOL or "eq 5d*" or "eq5d*" or "eq-5d*" or "short form 6d*" or "Short Form 6-Dimension" or "Short-Form 6-Dimension" or "Short-Form 6 Dimension" or "Short Form 6 Dimension" or "short-form 6d*" or "sf6d*" or "sf 6d*" or "sf-6d*" or "Health utilities index" or "HUI-2*" or "HUI2*" or "HUI-3*" or "HUI3*" or "EORTC-8D" or "AQoL*" or "EORTC-8D*" or "ICECAP*" or "QWB*")))) AND ((((method* or instrument* or tool* or measure* or statistic*) and (utilit* or (quality and life) or (patient and report*))) OR (((long* or extend*) and ("follow-up" or "follow up")) or "missing data") OR ((extrapolat* or regression) and (data or utilit*))))[Title]

# Inclusion criteria

The following types of articles, published in English, were eligible for inclusion:

- Methods papers detailing the analysis of patient level utility data including approaches for handling missing data, regression techniques and extrapolation methods
- Methods papers detailing how patient level utility data should be used within economic modelling
- Studies that compare the results of use of different methods to analyse patient-level utility data in cost-effectiveness models
- Simulation and validation studies that assess different statistical techniques using simulated or real-world patient-level utility datasets to compare their performance and validity
- HTA and industry body guidance documents reporting on how analysis of patient level utility data should be conducted

# PRISMA for literature review search

**Identification of new studies via other methods**

**Identification of new studies via databases and registers methods**

Records excluded

(n = 2254)

Records removed *before screening*:

Duplicate records removed

(n = 1001)

Records identified from:

Medline (n = 1672)

Embase (n = 1702)

PubMed Bookshelf (n = 5)

INAHTA (n = 17)

Records identified from:

Survey responses (n = 11)

Citation searching (n = 8)

Records identified from:

Targeted searches (n = 32) Expert knowledge (n = 2)

**Identification**

Records excluded

(n = 4)

Records screened at TIAB

(n = 19)

Records screened at TIAB

(n = 2395)

**Screening**

Records excluded

Wrong study design (n = 113)

Systematic review* (n = 16)

Wrong publication type (n = 4)

Wrong outcome (n = 2)

Foreign language (n = 1)

Records screened at full text

(n = 156)

* Systematic reviews identified during screening were excluded from synthesis but were used to mine references for eligible studies

Total studies included in literature review (n = 20)

**Included**

Total studies used in guidance development (n = 54)

# PRISMA for previous TA search

**Identification of previous TAs**

**Screening**

**Included**

**Identification**

Records identified from NICE website (n = 289)

Records excluded

- TA did not include utility values(n=51)
- Terminated appraisal (n=52)
- Patient level utility data not analysed (n=57)
- TA documents unavailable (n=4)

Records screened

(n = 289)

Total studies included in review

(n = 125)

# Scoping Survey

A scoping survey was conducted in March 2025 to 1) check whether the project team’s initial draft list of issues was complete and 2) to rank the issues in order of importance. The survey was advertised on the following channels:

•	The InterTASC network

•	The ABPI mailing list

•	The Health Economics distribution list at JISC mail

•	The PenTAG LinkedIn page

The survey was also shared directly with contacts at NICE and with Project Panel members.

In addition to filling out the survey, respondents were also asked to share any relevant studies they were aware of and relevant analysis code. Respondents were required to fill in details of which organisation they worked at and their primary methodological discipline, to ensure that we surveyed a cross section of people from across the field.

# Landscape review: Sifting and data extraction

All articles were collected in Endnote where they were deduped, before being moved into Rayyan for screening. Articles were screened at title and abstract by the lead reviewer (MSB) with a 10% sample independently reviewed by a second reviewer (CF). Any disagreements were resolved by discussion between the two reviewers and a third reviewer (DL). Where any patterns of disagreement were identified, these were resolved across all articles (not just the 10%). Full texts were screened by the lead reviewer (MSB), with a minimum 50% sample independently reviewed by a second reviewer (EN). Any disagreements were resolved by discussion between the two reviewers and a third reviewer (FGJ). Where any patterns of disagreement were identified, these were resolved across all articles (not just the 50%).

Data extraction was conducted using a standardised form. The following information was extracted: bibliographic details; disease area; type of study; issues considered; methods considered; recommendations. All studies were data extracted by the lead reviewer (MSB) with a 50% sample independently extracted by a second reviewer (EN). Any disagreements were resolved by discussion between the two reviewers and a third reviewer (FGJ).

# Targeted searches: methods detail

Methodological studies that covered the following issues/methods raised in the stakeholder surveys and/or as key issues in the NICE TA review were identified via pragmatic, targeted searches. Searches were performed in Google Scholar, Perplexity.AI and Claude.AI. Searches in Google Scholar used combinations of simple terms (examples provided below for each issue/method), followed by on-screen review of “cited” or “related articles”, when a potentially relevant article was identified. AI searches were initiated using prompts such as “You are performing a rapid review of methodological approaches to calculate “time to death” utilities in patient level utility analysis. Can you please identify up to ten key papers that cover this topic, provide their full citations, and give a brief summary?”. Again, these were removed on screen and “cited” or “related articles” of relevant articles were reviewed in Google Scholar. Because the search and sift was performed on-screen, articles that had already been retrieved in the main landscape review were excluded.

- Time to death utilities – e.g. (“time to death” OR TDD) AND utilit*
- Covariate selection in the context of utility analysis – e.g. covariate AND selection AND “utility analysis”
- Missing not at random (MNAR) methods for handling missing data in the context of utility analysis – e.g. (“missing not at random” OR MNAR) AND “utility analysis”
- Mixed model repeated measures (MMRM) methods for utility analysis – e.g. (“mixed model repeated measures” OR MMRM) AND “utility analysis”
- Meta-analysis / pooling of patient level utility data – e.g. (meta-analysis OR pooling) AND (patient OR individual) AND utilit*
- Analysis of utility data for fluctuating conditions – e.g. fluctuati* AND “utility analysis”
- Calculating adverse event multipliers or decrements from patient level utility data – e.g. adverse AND (multipl* OR decrement*) AND “utility analysis”
- Methods to account for floor and ceiling effects in analysis of utility data – e.g. (floor OR ceiling AND “utility analysis”)
- Utilities higher than the general population – e.g. (high OR excess) AND general population AND “utility analysis” AND methods

# TA review methods detail

TAs that included patient level analysis of utility data were included (those that did not contain such analysis were excluded). All TAs were screened by a single reviewer (SS). TAs were first screened to identify whether they implemented a cost-utility analysis approach, and then to identify whether they used individual patient utilities. Included records were extracted by a single reviewer (SS) with a 10% sample independently extracted by a second reviewer (FGJ). Any disagreements were resolved by discussion between the two reviewers and a third reviewer (DL).

# TA review results tables

Table 1: TA characteristics

| Characteristic | Yes % (n) | No % (n) |
| --- | --- | --- |
| Cancer appraisal (Y/N) | 60% (76) | 40% (49) |
| Rare disease (Y/N) | 30% (38) | 70% (87) |
| Fluctuating condition (Y/N) | 29% (36) | 71% (89) |
| Trial data source (Y/N) | 95% (119) | 5% (6) |
| Redacted utilities (Y/N) | 67% (84) | 33% (41) |
| Tariff reported (Y/N) | 41% (51) | 59% (74) |
| DSA method reported (Y/N) | 49% (61) | 51% (64) |
| PSA method reported (Y/N) | 46% (58) | 54% (67) |

Table 2: Utility data collection tools

| Utility data collection tool | Frequency |
| --- | --- |
| EQ-5D-5L | 73 (58%) |
| EQ-5D-3L | 24 (19%) |
| EORTC-QLQ-C30 | 9 (7%) |
| MSQv2 | 3 (2%) |
| MF-8D | 2 (2%) |
| ESS | 2 (2%) |
| SF-36 | 2 (2%) |
| UFS-QoL | 2 (2%) |
| 5-D Itch | 1 (1%) |
| ACQ-6 | 1 (1%) |
| CFQ-R 8D | 1 (1%) |
| EPP-QoL | 1 (1%) |
| FACT-MM | 1 (1%) |
| ISI | 1 (1%) |
| PROMIS-GH | 1 (1%) |
| WOMAC | 1 (1%) |
| Total | 125 |

Table 3: Timepoints of utility data collection

| Timepoints | Cancer | Non-cancer |
| --- | --- | --- |
| Less than 3 times per study period | 2 (3%) | 8 (17%) |
| 3 to 5 times per study period | 4 (5%) | 16 (35%) |
| More than 5 times per study period | 69 (92%) | 19 (41%) |
| One off timepoint | 0 (0%) | 3 (7%) |
| Total | 75 | 46 |

Note: Three non-cancer TAs did not report timepoints in the NICE committee papers and one cancer TA did not report timepoints in the NICE committee papers

Table 4: Follow up utility data collection in cancer TAs

| Post progression and follow up data collection | Number of TAs |
| --- | --- |
| ≥ one year post progression follow-up and ≥ 12 weekly measurement frequency | 10 (14%) |
| ≥ one year post progression follow-up and < 12 weekly measurement frequency | 4 (5%) |
| < one year post progression follow-up and ≥ 12 weekly measurement frequency | 10 (14%) |
| < one year post progression follow-up and < 12 weekly measurement frequency | 3 (4%) |
| Only one visit post progression or post discontinuation | 45 (63%) |
| Total reported post progression follow up data collection timepoints | 72 |

Note: Four TAs did not report follow up data collection timepoints in NICE committee papers

Table 5: Health states reported in TAs

| Health State | Cancer | Non-cancer |
| --- | --- | --- |
| Progression status | 51 (68%) | 3 (7%) |
| Functional status | 0 (0%) | 33 (67%) |
| Time to death status | 9 (12%) | 0 (0%) |
| Treatment status | 3 (4%) | 5 (10%) |
| Response status | 3 (4%) | 6 (12%) |
| Timepoint | 0 (0%) | 2 (4%) |
| Recurrence or event free survival | 9 (12%) | 0 (0%) |
| Total reported health states | 75 | 49 |

# Narrative description of key themes in Review #1 (landscape review)

## Theme 1: Missing data

For missing data, it was noted by Bandos et al. (2025)^1^ that there is currently no gold standard where data are missing not at random (MNAR), although another study (Anon, 2005)^2^ showed that in this situation, analyses that assume missing at random (MAR) tend to underestimate quality of life decline compared to approaches assuming MNAR either based on early withdrawal for any reason or taking account of death for any reason.

Complementing these two studies on cancer, in the neurological sphere, Belger et al. (2016)^3^ found that multiple imputation (MI) is preferable where data are MAR, although if the missing completely at random (MCAR) assumption is met, naïve methods perform just as well. Blough et al. (2009)^4^ found that imputation methods had a substantial impact on the ICER and uncertainty in a respiratory trial, noting that conservative single imputation resulted in less uncertainty. Indeed, Faria et al. (2014)^5^ noted that MI is more complex and can be less efficient than single imputation, although likelihood models can be an alternative to MI, and where missing data are monotonic, inverse probability weights (IPW) methods can be useful.

In musculoskeletal conditions, Kaplan et al. (2005)^6^ showed that while imputed data were significantly correlated with observed data, they had substantial limitations including constrained variability and poorer prediction of clinical benchmarks. In the study by Manca et al. (2005)^7^ the impact of using different imputation methods was not consistent across the examples. Michalowsky et al. (2020)^8^ found that item imputation resulted in a much higher incremental cost effectiveness ratio (ICER) than aggregate imputation in a simulation study conducted alongside a cluster-randomised controlled trial in the field of dementia. Furthermore, Hunter et al found that multiple imputation overcame the severe limitations of other methods to handling missing data, including last observation carried forward. However, Simons et al. (2015)^9^ found that the most effective type of imputation depended on the sample size and nature of missing data in a simulation study conducted alongside a randomised controlled trial of neurosurgery.

## Theme 2: Regression

Griffiths et al. (2017)^10^ showed the value of a mixed regression model in explaining variation in EQ-5D data according to key clinical outcomes and patient characteristics. Kaproulia et al. (2022) found that random effects mediation models (RMME) were best in right-bounded and multimodality datasets. The linear mixed model was best in the left-bounded dataset. Kaproulia et al. (2024) found that in the event of post-progression drop, this was underestimated by RMME. Considering the value of regression analysis, Briggs et al. (2016)^11^ found that as trials are rarely powered to detect differences in health-related quality of life, a pre-specified regression analysis may be acceptable to improve the precision of the effects and understand their origin. Meanwhile Hunter et al. (2015)^12^ showed that linear regression with baseline adjustment is a recommended adjustment method in regression modelling.

## Theme 3: Covariate selection

Wittenberg et al. (2017)^13^ found that the minimum estimator often performed best in covariate selection. Manca et al found that widely used methods to calculate diﬀerential QALYs may provide incorrect results in the presence of baseline mean utility imbalance regardless of whether these diﬀerences are formally statistically signiﬁcant. Multiple regression methods can be usefully applied to generate appropriate estimates of diﬀerential mean QALYs and an associated measure of sampling variability, while controlling for diﬀerences in baseline mean utility between treatment arms in the trial.

## Theme 4: Other issues

A few further issues were considered by single studies. Bojke et al. (2017)^14^ noted that there is currently no guidance on extrapolating costs and utilities in the context of short-term data, and little justification for approaches taken, although approaches taken to temporal extrapolation may be too simplistic. Pandey et al. (2022)^15^ found that in handling data with heavy ceiling effects, the log-transformed two-part model with ordinary least squares (OLS) was best followed by the log-transformed two-part model with tobit. Hill et al. (2019)^16^ found that over half of appraisals applied some form of age adjustment. Committees rarely commented on the limitations of using age-adjusted adult utilities for paediatric populations. While the authors encouraged use of paediatric specific utility measures, where adult measures were to be used, it was recommended that an exchange rate could be agreed for adjustment of utilities.

HTA bodies searched
The full list of HTA bodies searched were:

- National Institute for Health and Care Excellence (NICE)
- NICE Decision Support Unit (University of Sheffield)
- Scottish Medicines Consortium (SMC)
- Canada's Drug Agency (CDA-AMC)
- Institute for Clinical and Economic Review (ICER)
- Pharmaceutical Benefits Advisory Committee (PBAC)
- National Health Care Institute (ZIN)
- Haute Autorité de Santé (HAS)
- Guidance for the new Joint Clinical Assessments (European Union)

# List of studies excluded from the systematic review

| First author and year | Title | Reason for exclusion |
| --- | --- | --- |
| Abaci 2012 | Long term cost-effectiveness analysis of ticagrelor in patients with acute coronary syndromes from a turkish health care perspective based on data from the plato trial | Wrong study design |
| Adams 2024 | On the Trade-Off between Fidelity, Utility and Privacy of Synthetic Patient Data | Wrong study design |
| Arnold 2009 | Comparison of direct and indirect methods of estimating health state utilities for resource allocation: Review and empirical analysis | Wrong study design |
| Badia 2018 | Mapping AcroQoL scores to EQ-5D to obtain utility values for patients with acromegaly | Wrong study design |
| Baio 2014 | Bayesian models for cost-effectiveness analysis in the presence of structural zero costs | Wrong study design |
| Bellamy 1996 | Measurement of patient-derived utility values for periodontal health using a multi-attribute scale | Wrong study design |
| Betts 2020 | Utility value estimates in cardiovascular disease and the effect of changing elicitation methods: a systematic literature review | Wrong publication type (systematic review) |
| Blumenschein 1998 | Relationship between quality of life instruments, health state utilities, and willingness to pay in patients with asthma | Wrong study design |
| Bottomley 2003 | Health-related quality of life in non-small-cell lung cancer: methodologic issues in randomized controlled trials | Wrong publication type (systematic review) |
| Boye 2014 | Challenges to time trade-off utility assessment methods: when should you consider alternative approaches? | Wrong study design |
| Brauer 2006 | Trends in the measurement of health utilities in published cost-utility analyses | Wrong publication type (systematic review) |
| Breckenridge 2015 | How to routinely collect data on patient-reported outcome and experience measures in renal registries in Europe: an expert consensus meeting | Wrong study design |
| Breslin 1991 | Quality of life: how is it measured and defined? | Wrong study design |
| Brinsmead 2003 | Use of pharmacoeconomics in prescribing research. Part 4: is cost-utility analysis a useful tool? | Wrong study design |
| Buchholz 2006 | Utility assessment to measure the impact of dry eye disease | Wrong study design |
| Bulamu 2024 | Measurement properties of utility-based health-related quality of life measures in cardiac rehabilitation and secondary prevention programs: a systematic review | Wrong publication type (systematic review) |
| Callahan 2022 | Measures of Utility Among Studies of Genomic Medicine for Critically Ill Infants: A Systematic Review | Wrong publication type (systematic review) |
| Calsavara 2023 | Simulation study comparing analytical methods for single-item longitudinal patient-reported outcomes data | Wrong outcome |
| Carlson 2015 | Economic evaluation of tocilizumab monotherapy compared to adalimumab monotherapy in the treatment of severe active rheumatoid arthritis | Wrong study design |
| Carreno 2011 | Using HAQ-DI to estimate HUI-3 and EQ-5D utility values for patients with rheumatoid arthritis in Spain | Wrong study design |
| Chan 2010 | Cost-effectiveness of cetuximab combined with radiotherapy for patients with locally advanced head and neck cancer in Taiwan | Wrong study design |
| Chen 2009 | Cost-effectiveness analysis of telbivudine vs lamivudine in treating the patients with hbeag-positive and -negative chronic hepatitis B in China | Wrong study design |
| Chen 2018 | INOTUZUMAB OZOGAMICIN IN PATIENTS WITH RELAPSED OR REFRACTORY (R/R) ACUTE LYMPHOCYTIC LEUKEMIA (ALL): ESTABLISHING A VALUE-BASED COST - PRELIMINARY RESULTS | Wrong study design |
| Cheung 2018 | A Novel, Simple and Accurate Method to Map Quality-Of-Life Scores to Utility Values | Wrong study design |
| Chouaid 2017 | Cost-Effectiveness Analysis of Afatinib versus Gefitinib for First-Line Treatment of Advanced EGFR-Mutated Advanced Non-Small Cell Lung Cancers | Wrong study design |
| Clay 2011 | Predicting EQ-5D utility scores from the huntington quality of life instrument (H-QOL-I) | Wrong study design |
| Cortes 2009 | Cost-effectiveness analyses of docetaxel versus paclitaxel once weekly in patients with metastatic breast cancer progressed after anthracycline chemotherapy in Spain | Wrong study design |
| Cunningham 2003 | A cost-utility analysis of patients undergoing orthognathic treatment for the management of dentofacial disharmony | Wrong study design |
| Czoski 2014 | A utility algorithm forthe pressure ulcer quality of life-utility instrument (PUQol-UI) | Wrong study design |
| Dakin 2018 | Review and critical appraisal of studies mapping from quality of life or clinical measures to EQ-5D: an online database and application of the MAPS statement | Wrong publication type (systematic review) |
| de Freitas 2022 | PCR93 Improving the Methods of Vignette Development for Utility Estimation: A Case Study in Pompe Disease | Wrong study design |
| Del Hoyo 2019 | Telemonitoring of Crohn's Disease and Ulcerative Colitis (TECCU): Cost-Effectiveness Analysis | Wrong study design |
| Diamantopoulos 2009 | Cost-effectiveness of tocilizumab compared to standard of care for the treatment of moderate/severe rheumatoid arthritis (RA) in Italy | Wrong study design |
| Dolan 1998 | The measurement of individual utility and social welfare | Wrong study design |
| Earnshaw 2009 | Cost-effectiveness of patient selection using penumbral-based MRI for intravenous thrombolysis | Wrong study design |
| Eekhout 2014 | Missing data in a multi-item instrument were best handled by multiple imputation at the item score level | Wrong outcome |
| Ertenli 2012 | Cost-effectiveness of tocilizumab for the treatment of active rheumatoid arthritis (RA) patients with inadequate response to anti-TNF treatment in Turkey | Wrong study design |
| Espinosa 2014 | Cost-utility analysis of pazopanib versus sunitinib as first-line treatment of metastatic renal cell carcinoma (mRCC) in Spain | Wrong study design |
| Etges 2022 | Telemedicine Versus Face-to-Face Care in Ophthalmology: Costs and Utility Measures in a Real-World Setting | Wrong study design |
| Fawsitt 2019 | Choice of Prosthetic Implant Combinations in Total Hip Replacement: Cost-Effectiveness Analysis Using UK and Swedish Hip Joint Registries Data | Wrong study design |
| Feldman 2020 | Cost-effectiveness of brentuximab vedotin with chemotherapy in treatment of CD30-expressing PTCL | Wrong study design |
| Finnell 2012 | Application of classic utilities to published pediatric cost-utility studies | Wrong publication type (systematic review) |
| Franken 2016 | External validity at stake in mapping non-preference based disease specific measures to generic preference-based utility values in advanced cutaneous melanoma | Wrong study design |
| Fryback 2001 | Bayesian cost-effectiveness analysis. An example using the GUSTO trial | Wrong study design |
| Gagnon 2011 | A novel way of estimating cost-effectiveness ratios from clinical trials with missing data: A simulation study | Wrong publication type (abstract) |
| Gamst-Klaussen 2018 | Assessment of outcome measures for cost-utility analysis in depression: mapping depression scales onto the EQ-5D-5L | Wrong study design |
| Gillard 2010 | Mapping from disease-specific measures to health-state utility values in chronic migraineurs | Wrong study design |
| Gillard 2012 | Mapping from disease-specific measures to health-state utility values in individuals with migraine | Wrong study design |
| Glasziou 2007 | Which health-related quality of life score? A comparison of alternative utility measures in patients with Type 2 diabetes in the ADVANCE trial | Wrong study design |
| Goncalves 2023 | Mapping studies to estimate health-state utilities from nonpreference-based outcome measures: a systematic review on how repeated measurements are taken into account | Wrong publication type (systematic review) |
| Goossens 1999 | Patient utilities in chronic musculoskeletal pain: how useful is the standard gamble method? | Wrong study design |
| Gourzoulidis 2018 | Cost-Effectiveness of Empagliflozin for the Treatment of Patients with Type 2 Diabetes Mellitus at Increased Cardiovascular Risk in Greece | Wrong study design |
| Gout-Zwart 2017 | Cost-effectiveness analysis of a point-of-care test for renal function measurement in community pharmacies in the Netherlands to prevent antibiotic-related hospitalizations | Wrong publication type (abstract) |
| Gray 2018 | Development of Methods for the Mapping of Utilities Using Mixture Models: Mapping the AQLQ-S to the EQ-5D-5L and the HUI3 in Patients with Asthma | Wrong study design |
| Hanita 2000 | Self-report measures of patient utility: should we trust them? | Wrong study design |
| Hernandez Alava 2014 | A comparison of direct and indirect methods for the estimation of health utilities from clinical outcomes | Wrong study design |
| Hill 2020 | A Review of the Methods Used to Generate Utility Values in NICE Technology Assessments for Children and Adolescents | Wrong publication type (systematic review) |
| Hirt 2019 | Cost Effectiveness of the Third-Generation Tyrosine Kinase Inhibitor (TKI) Ponatinib, vs. Second-Generation TKIs or Stem Cell Transplant, as Third-Line Treatment for Chronic-Phase Chronic Myeloid Leukemia | Wrong study design |
| Hudgens 2014 | Comparison of methods to estimate health state utilities in metastatic breast cancer (MBC) | Wrong study design |
| Hussain 2016 | Validity of standard gamble utilities in patients referred for aortic valve replacement | Wrong study design |
| Huynh 2017 | Values for the ICECAP-Supportive Care Measure (ICECAP-SCM) for use in economic evaluation at end of life | Wrong study design |
| Ismaila 2014 | COST-effectiveness of salmeterol/fluticasone propionate combination (Advair®) in uncontrolled asthma in Canada | Wrong study design |
| Karimi 2019 | Effect of Reflection and Deliberation on Health State Values: A Mixed-Methods Study | Wrong study design |
| Katanyoo 2022 | A comparison of six approaches for measuring utility values among patients with locally advanced cervical cancer | Wrong study design |
| Kharroubi 2015 | Use of Bayesian Markov chain Monte Carlo methods to estimate EQ-5D utility scores from EORTC QLQ data in myeloma for use in cost-effectiveness analysis | Wrong study design |
| Khurana 2017 | Assessing methodologies for health state utilities in paediatric indications for cost-utility analyses: Review of nice technology appraisals for paediatric indications | Wrong publication type (abstract) |
| Kiebert 1993 | Test-retest reliabilities of two treatment-preference instruments in measuring utilities | Wrong study design |
| Kitchener 2014 | The clinical effectiveness and cost-effectiveness of primary human papillomavirus cervical screening in England: extended follow-up of the ARTISTIC randomised trial cohort through three screening rounds | Wrong study design |
| Kong 1997 | Methodologic assessments of quality of life measures in clinical trials | Wrong publication type (systematic review) |
| Konig 2009 | Utility assessment in patients with mental disorders: validity and discriminative ability of the time trade-off method | Wrong study design |
| Kotirum 2017 | A cost-utility analysis of dabigatran, enoxaparin, and usual care for venous thromboprophylaxis after hip or knee replacement surgery in Thailand | Wrong study design |
| Kuspinar 2013 | Do generic utility measures capture what is important to the quality of life of people with multiple sclerosis? | Wrong study design |
| Lacey 2015 | Quality of Life and Utility Measurement in a Large Clinical Trial Sample of Patients with Mild to Moderate Alzheimer's Disease: Determinants and Level of Changes Observed | Wrong study design |
| Lee 2017 | Health utility in myelofibrosis patients and comparison between health utility values measured by time trade off and visual analogue scale | Wrong study design |
| Lee 2023 | Bayesian treatment screening and selection using subgroup-specific utilities of response and toxicity | Wrong study design |
| Leeyaphan 2011 | Measuring melasma patients' quality of life using willingness to pay and time trade-off methods in Thai population | Wrong study design |
| Liew 2017 | Cost utility analysis (CUA) of nivolumab compared to everolimus for the treatment of metastatic rena L cell carcinoma (RCC) in Australia | Wrong study design |
| Lin 2017 | Cost-effectiveness of PET-CT versus adrenal vein sampling for the diagnosis of hypertension caused by primary aldosteronism | Wrong study design |
| Lloyd 2018 | HOW DO WE MEASURE UTILITIES OUTSIDE OF TRIALS IN RARE DISEASES? | Wrong study design |
| Lou 2011 | Utilizing a paper standard gamble instrument to assess health utility in patients with hemophilia B | Wrong study design |
| Luo 2024 | Cost-utility analysis of romiplostim for the treatment of chronic primary immune thrombocytopenia in China | Wrong study design |
| Maor 2001 | A comparison of three measures: the time trade-off technique, global health-related quality of life and the SF-36 in dialysis patients | Wrong study design |
| Marsh 2014 | Model-based cost-effectiveness analyses for the treatment of chronic lymphocytic leukaemia: a review of methods to model disease outcomes and estimate utility | Wrong publication type (systematic review) |
| Matito 2013 | Serum Tryptase Monitoring in Indolent Systemic Mastocytosis: Association with Disease Features and Patient Outcome | Wrong study design |
| McGrath 2010 | Using qualitative methods to inform the trade-off between content validity and consistency in utility assessment: the example of type 2 diabetes and Alzheimer's disease | Wrong publication type (systematic review) |
| McTaggart-Cowan 2013 | Mapping utilities from cancer-specific health-related quality of life instruments: a review of the literature | Wrong publication type (systematic review) |
| Mercer 2016 | The CARE Plus study - a whole-system intervention to improve quality of life of primary care patients with multimorbidity in areas of high socioeconomic deprivation: exploratory cluster randomised controlled trial and cost-utility analysis | Wrong study design |
| Mihalopoulos 2014 | Assessing outcomes for cost-utility analysis in depression: comparison of five multi-attribute utility instruments with two depression-specific outcome measures | Wrong study design |
| Mitsakakis 2016 | Investigating structural independence in preference-based utility instruments using graphical models | Wrong study design |
| Msaouel 2024 | Risk-benefit trade-offs and precision utilities in phase I-II clinical trials | Wrong study design |
| Mujica-Mota 2017 | Deriving mapped health state utility values for economic evaluation using summary health related quality of life measures as sufficient statistics | Wrong study design |
| Mukherjee 2023 | Handling Missing Data in Health Economics and Outcomes Research (HEOR): A Systematic Review and Practical Recommendations | Wrong publication type (systematic review) |
| Omar Alsaleh 2023 | MSR120 A Method for Adjusting US Health State Utilities for Use in the UK | Wrong study design |
| Peiris 2020 | Mapping the Functional Independence Measure to a multi-attribute utility instrument for economic evaluations in rehabilitation: a secondary analysis of randomized controlled trial data | Wrong study design |
| Perez 1997 | A comparison of time trade-off and quality of life measures in patients with advanced cancer | Wrong study design |
| Petrillo 2010 | Employing multi-attribute utility theory to develop the exact-u: A preference-based measure for copd exacerbation utilities | Wrong study design |
| Pickard 2009 | A patient-based utility measure of health for clinical trials of cancer therapy based on the European Organization for the Research and Treatment of Cancer Quality of Life Questionnaire | Wrong study design |
| Pilz 2024 | Cancer-Specific Health Utilities: Evaluation of Core Measurement Properties of the EORTC QLU-C10D in Lung Cancer Patients-Data from Four Multicentre LUX-Lung Trials, Applying Six Country Tariffs | Wrong study design |
| Prosser 2012 | Health utility elicitation: Is there still a role for direct methods? | Wrong study design |
| Ramaekers 2013 | Cost effectiveness of modified fractionation radiotherapy versus conventional radiotherapy for unresected non-small-cell lung cancer patients | Wrong study design |
| Rankin 2018 | Methods used to value health related quality in a systematic review of cost-utility analyses in heart failure | Wrong study design |
| Richardson 2012 | The development of a model for translation of the Neck Disability Index to utility scores for cost-utility analysis in cervical disorders | Wrong study design |
| Richardson 2015 | Why do multi-attribute utility instruments produce different utilities: the relative importance of the descriptive systems, scale and 'micro-utility' effects | Wrong study design |
| Richardson 2015 | Comparing and explaining differences in the magnitude, content, and sensitivity of utilities predicted by the EQ-5D, SF-6D, HUI 3, 15D, QWB, and AQoL-8D multiattribute utility instruments | Wrong study design |
| Roberts 2009 | Utility measures of health-related quality of life in patients treated for benign paroxysmal positional vertigo | Wrong study design |
| Roe 2022 | The utility of patient-reported outcome measures in mental health | Wrong study design |
| Rui 2022 | Cost-effectiveness analysis of sintilimab + chemotherapy versus camrelizumab + chemotherapy for the treatment of first-line locally advanced or metastatic nonsquamous NSCLC in China | Wrong study design |
| Sarioz 2017 | The cost-effectiveness of sacubitril/valsartan in the treatment of chronic heart failure with reduced ejection fraction in turkey | Wrong study design |
| Serra-Burriel 2019 | Transient elastography for screening of liver fibrosis: Cost-effectiveness analysis from six prospective cohorts in Europe and Asia | Wrong study design |
| Shah 2004 | TTO utility scores measure quality of life in patients with visual morbidity due to diabetic retinopathy or ARMD | Wrong study design |
| Sharma 2002 | Validity of the time trade-off and standard gamble methods of utility assessment in retinal patients | Wrong study design |
| Shaw 2021 | A Comparison of Generic and Condition-Specific Preference-Based Measures Using Data From Nivolumab Trials: EQ-5D-3L, Mapping to the EQ-5D-5L, and European Organisation for Research and Treatment of Cancer Quality of Life Utility Measure-Core 10 Dimensions | Wrong study design |
| Shen 2019 | Using Time Trade-Off Methods to Elicit Short-Term Utilities Associated with Treatments for Bulbar Urethral Stricture | Wrong study design |
| Shimbo 2011 | Cost-effectiveness of peginterferon and ribavirin for elderly patients with chronic hepatitis C: Results based on the nationwide hepatitis registration in Japan | Wrong study design |
| Slof 2012 | Cost-effectiveness analysis of early versus non-early intervention in acute migraine based on evidence from the 'Act when mild' study | Wrong study design |
| Sullivan 2016 | Measurement of utility in asthma: evidence indicating that generic instruments may miss clinically important changes | Wrong study design |
| Szafranski 2024 | The Determination of Diabetes Utilities, Costs, and Effects Model: A Cost-Utility Tool Using Patient-Level Microsimulation to Evaluate Sensor-Based Glucose Monitoring Systems in Type 1 and Type 2 Diabetes: Comparative Validation | Wrong study design |
| Szende 2010 | Measurement of utility values in the UK for health states related to immune thrombocytopenic purpura | Wrong study design |
| Tijhuis 2000 | Value of the time trade off method for measuring utilities in patients with rheumatoid arthritis | Wrong study design |
| Tikhonova 2016 | Azacitidine for treating acute myeloid Leukaemia: A nice single technology appraisal | Wrong study design |
| Van Nooten 2009 | Economic evaluation of ezetimibe combined with simvastatin for treatment of primary hypercholesterolaemia | Wrong study design |
| Vargas 2016 | Cost effectiveness of nivolumab for the treatment of metastatic melanoma in Chile | Wrong publication type (abstract) |
| Ward 2017 | Applicability of patient utilities as measures of overall quality of life in rheumatoid arthritis clinical trials | Wrong study design |
| Ward Fuller 2017 | Health State Preference Weights for the Glasgow Outcome Scale Following Traumatic Brain Injury: A Systematic Review and Mapping Study | Wrong publication type (systematic review) |
| Wee 2018 | Mean Rank, Equipercentile, and Regression Mapping of World Health Organization Quality of Life Brief (WHOQOL-BREF) to EuroQoL 5 Dimensions 5 Levels (EQ-5D-5L) Utilities | Wrong study design |
| Whately-Smith 2014 | Utility values in health technology assessments: a statistician’s perspective | Wrong study design |
| Williams 2017 | Estimation of Survival Probabilities for Use in Cost-effectiveness Analyses: A Comparison of a Multi-state Modeling Survival Analysis Approach with Partitioned Survival and Markov Decision-Analytic Modeling | Wrong study design |
| Wu 2015 | Examining the impact of 11 long-standing health conditions on health-related quality of life using the EQ-5D in a general population sample | Wrong study design |
| Xie 2009 | Results of a model analysis to estimate cost utility and value of information for intravenous immunoglobulin in Canadian adults with chronic immune thrombocytopenic purpura | Wrong study design |
| Yepes-Nunez 2011 | Preferences of states of health and measurements of utility | Foreign language paper |
| Yong 2014 | The Measurement Of Utilities In Asthma Patients: A Preliminary Study | Wrong study design |
| Yong 2016 | Development, feasibility, and validity of a computer-based utility assessment tool for measuring asthma-specific health utilities in Malaysia using the standard gamble method | Wrong study design |
| Zhang 2024 | Generating Utilities for the Chateau-Sante Base: A Novel, Generic, and Patient-Centered Health-Outcome Measure | Wrong study design |
| Zhou 2025 | Health utilities used in oncology cost-utility analyses: a registry-based analysis | Wrong publication type (systematic review) |
| Zhu 2014 | Cost-effectiveness analysis of computed tomographic colonography versus double contrast barium enema for investigation of patients with symptoms of colorectal cancer: Economic evaluation alongside the SIGGAR trial | Wrong study design |
| Zhu 2020 | Cost-effectiveness of pembrolizumab plus axitinib as first-line therapy for advanced renal cell carcinoma | Wrong study design |

# Included TAs

TA Number

127

540

688

737

758

763

765

767

768

769

772

773

775

777

778

779

781

786

787

788

789

791

792

801

807

810

813

816

817

818

819

825

827

830

832

833

837

849

851

853

854

857

858

860

862

865

866

870

874

876

877

880

881

882

886

888

890

891

892

893

895

902

904

906

907

909

911

913

914

915

917

921

922

926

927

929

930

934

935

939

944

946

947

948

949

950

951

952

954

955

957

959

962

964

967

970

973

974

979

983

986

988

989

992

993

995

996

997

1000

1001

1005

1008

1009

1010

1011

1012

1014

1015

1017

1018

1023

HST19

HST25

HST27

HST28

# Excluded TAs

TA Number

155

274

283

298

312

599

616

755

757

764

766

770

771

774

776

780

782

783

784

785

790

793

794

795

796

797

798

799

800

802

803

804

805

806

808

809

811

812

814

815

820

821

822

823

824

826

828

829

835

836

838

839

840

841

842

843

844

845

846

847

848

850

852

856

859

861

863

864

867

868

871

872

873

875

878

879

883

884

887

889

894

896

897

898

899

900

901

903

905

908

910

912

916

918

919

920

923

924

925

928

931

932

933

936

937

938

940

941

942

943

945

953

956

958

960

961

963

965

966

968

969

971

972

975

976

977

978

980

982

984

985

987

990

991

994

998

999

1002

1003

1004

1006

1007

1013

1016

1019

1020

1021

1022

1024

1025

1026

HST15

HST17

HST18

HST20

HST21

HST22

HST23

HST24

HST26

HST29

HST30

HST31

HST8

# References

1. Bandos H, Torres-Saavedra PA, Culakova E, Gunn HJ, Lee MK, Duan F, et al. Best practices and pragmatic approaches for patient-reported outcomes and quality of life measures in cancer clinical trials. Journal of the National Cancer Institute Monographs. 2025;2025(68):14-21.

2. Anonymous. Benefits of epoetin alfa for cancer patients' quality of life are confirmed after modelling to account for missing data. Current medical research and opinion. 2005;21:S6-8.

3. Belger M, Haro JM, Reed C, Happich M, Kahle-Wrobleski K, Argimon JM, et al. How to deal with missing longitudinal data in cost of illness analysis in Alzheimer's disease-suggestions from the GERAS observational study. BMC Med Res Methodol. 2016;16:83.

4. Blough DK, Ramsey S, Sullivan SD, Yusen R. The impact of using different imputation methods for missing quality of life scores on the estimation of the cost-effectiveness of lung-volume-reduction surgery. Health Econ. 2009;18(1):91-101.

5. Faria R, Gomes M, Epstein D, White IR. A guide to handling missing data in cost-effectiveness analysis conducted within randomised controlled trials. Pharmacoeconomics. 2014;32(12):1157-70.

6. Kaplan RM, Groessl EJ, Sengupta N, Sieber WJ, Ganiats TG. Comparison of measured utility scores and imputed scores from the SF-36 in patients with rheumatoid arthritis. Med Care. 2005;43(1):79-87.

7. Manca A, Palmer S. Handling missing data in patient-level cost-effectiveness analysis alongside randomised clinical trials. Appl Health Econ Health Policy. 2005;4(2):65-75.

8. Michalowsky B, Hoffmann W, Kennedy K, Xie F. Is the whole larger than the sum of its parts? Impact of missing data imputation in economic evaluation conducted alongside randomized controlled trials. Eur J Health Econ. 2020;21(5):717-28.

9. Simons CL, Rivero-Arias O, Yu LM, Simon J. Multiple imputation to deal with missing EQ-5D-3L data: Should we impute individual domains or the actual index? Qual Life Res. 2015;24(4):805-15.

10. Griffiths A, Paracha N, Davies A, Branscombe N, Cowie MR, Sculpher M. Analyzing Health-Related Quality of Life Data to Estimate Parameters for Cost-Effectiveness Models: An Example Using Longitudinal EQ-5D Data from the SHIFT Randomized Controlled Trial. Adv Ther. 2017;34(3):753-64.

11. Briggs AH, Parfrey PS, Khan N, Tseng S, Dehmel B, Kubo Y, et al. Analyzing Health-Related Quality of Life in the EVOLVE Trial: The Joint Impact of Treatment and Clinical Events. Med Decis Making. 2016;36(8):965-72.

12. Hunter RM, Baio G, Butt T, Morris S, Round J, Freemantle N. An educational review of the statistical issues in analysing utility data for cost-utility analysis. Pharmacoeconomics. 2015;33(4):355-66.

13. Wittenberg E, Bray JW, Gebremariam A, Aden B, Nosyk B, Schackman BR. Joint Utility Estimators in Substance Use Disorders. Value Health. 2017;20(3):458-65.

14. Bojke L, Manca A, Asaria M, Mahon R, Ren S, Palmer S. How to Appropriately Extrapolate Costs and Utilities in Cost-Effectiveness Analysis. Pharmacoeconomics. 2017;35(8):767-76.

15. Pandey S, Singh B, Kaur S, Sharma A. MSR115 Comparison of Statistical Models to Address Ceiling Effect in Utility Scores: A Simulation Study. Value in Health. 2022;25(12):S372.

16. Hill H, Rowen D, Pennington B, Wong R, Wailoo A. A review of the methods used to estimate and model utility values in NICE technology appraisals for paediatric populations. Report by the Decision Support Unit. Sheffield: School of Health and Related Research, University of Sheffield; 2019. Available from: <https://www.sheffield.ac.uk/media/34049/download?attachment>.
